# No Evidence for a Culturable Bacterial Tetrodotoxin Producer in *Pleurobranchaea maculata* (Gastropoda: Pleurobranchidae) and *Stylochoplana* sp. (Platyhelminthes: Polycladida)

**DOI:** 10.3390/toxins7020255

**Published:** 2015-01-28

**Authors:** Lauren R. Salvitti, Susanna A. Wood, Paul McNabb, Stephen Craig Cary

**Affiliations:** 1 Department of Biological Sciences, University of Waikato, Private Bag 3105, Hamilton 3240, New Zealand; E-Mails: ls161@students.waikato.ac.nz (L.R.S.); susie.wood@cawthron.org.nz (S.A.W.); 2 Cawthron Institute, Nelson 7042, New Zealand; E-Mail: paul.mcnabb@cawthron.org.nz

**Keywords:** Tetrodotoxin, bacteria, liquid chromatography-mass spectrometry, *Pleurobranchaea maculata*, *Stylochoplana* sp.

## Abstract

Tetrodotoxin (TTX) is a potent neurotoxin found in the tissues of many taxonomically diverse organisms. Its origin has been the topic of much debate, with suggestions including endogenous production, acquisition through diet, and symbiotic bacterial synthesis. Bacterial production of TTX has been reported in isolates from marine biota, but at lower than expected concentrations. In this study, 102 strains were isolated from *Pleurobranchaea maculata* (Opisthobranchia) and *Stylochoplana* sp. (Platyhelminthes). Tetrodotoxin production was tested utilizing a recently developed sensitive method to detect the C9 base of TTX via liquid chromatography—mass spectrometry. Bacterial strains were characterized by sequencing a region of the 16S ribosomal RNA gene. To account for the possibility that TTX is produced by a consortium of bacteria, a series of experiments using marine broth spiked with various *P. maculata* tissues were undertaken. Sixteen unique strains from *P. maculata* and one from *Stylochoplana* sp. were isolated, representing eight different genera; *Pseudomonadales*, *Actinomycetales*, *Oceanospirillales*, *Thiotrichales*, *Rhodobacterales*, *Sphingomonadales*, *Bacillales*, and *Vibrionales*. Molecular fingerprinting of bacterial communities from broth experiments showed little change over the first four days. No C9 base or TTX was detected in isolates or broth experiments (past day 0), suggesting a culturable microbial source of TTX in *P. maculata* and *Stylochoplana* sp. is unlikely.

## 1. Introduction

Tetrodotoxin (TTX) is a small non-protein neurotoxin closely related to saxitoxin [1,2]. It selectively targets voltage-gated sodium channels, resulting in the inhibition of action potentials across neurons. Ingestion of quantities as little as 1–2 mg can be fatal to humans [3,4]. Its highly selective nature has resulted in its frequent use in neurological medical studies, yet its biosynthetic pathway is still largely unknown [5,6]. The name tetrodotoxin is derived from the *tetrodontidae* order of pufferfish, in which TTX was first found. However, it has since been discovered globally in a wide range of organisms covering eight different phyla, excluding bacteria [5]. The source of TTX and its distribution among so many phylogenetically unrelated species remains a mystery. The most commonly cited hypothesis is that TTX has a bacterial origin (Table 1). In 1986, the first TTX-producing bacteria, a *Pseudomonas* species, was isolated from a red calcareous alga, *Jania* sp. [7]. Tetrodotoxin and the TTX analogue anhydro-tetrodotoxin were detected via high performance liquid chromatography (HPLC) and mouse bioassay [7].

Tetrodotoxin producing bacteria representing 22 genera have since been isolated from a range of host organisms including; puffer fish, octopi, sea stars, reef crabs, sea urchins, sea snails, gastropods, worms, and algae [5,8,9,10]. A summary of the bacterial genera, the concentrations of TTX they produce, the method of detection, and the organisms they were isolated from is provided in Table 1. The most common method of bacterial isolation among these studies involves homogenization of the host organism tissue followed by plating of aliquots onto non-selective medium. Individual bacterial strains are then selected and cultured in liquid media before harvesting and testing for TTX via various methods including; mouse bioassay, enzyme-linked immunosorbent assay (ELISA), gas chromatography-mass spectrometry (GC-MS), and HPLC (Table 1) [11,12,13,14,15,16]. However, the TTX concentrations in these bacterial cultures are significantly lower than the amounts contained in host organisms leading to doubt that they are the definitive source of TTX [16,17,18,19]. For example, Wang *et al.* [15] reported a maximal TTX concentration of 184 ng·g^−1^ from an isolated *Vibrio* sp. in comparison to 36 μg·g^−1^ tissue in the host sea snail *Nassarius semiplicatus*.

Matsumura [20] provided additional uncertainty by demonstrating that the culture media used to isolate the TTX producing bacteria could produce false positives for TTX when analyzed by HPLC and GC-MS. Of the numerous studies demonstrating bacterial TTX-production, to our knowledge only one [21] has used liquid chromatography-mass spectrometry (LC-MS) to confirm the presence of TTX (Table 1). The use of non-disputable chemical methods as a means of quantifying TTX in bacterial isolates would greatly assist in dispelling the controversy surrounding the bacterial origin of TTX.

**Table 1:** Bacteria reported to produce tetrodotoxin (TTX) or TTX like compounds.

Ref	Source	Toxicity of Host Species/Tissue *	Bacteria	Toxicity (TTX or Related Substances) **	Detection Method *
[10]	*Takifugu niphobles* (pufferfish)	intestines: N/A	*Raoultella terrigena*	4.3 μg·L^−1^	ELISA
[22]	*Fugu obscurus* (pufferfish)	liver: 80 MU·g^−1^	*Lysinibacillus fusiformis*	23.9 MU in 200 mL broth	mouse bioassay
[14]	*Fugu obscurus* (pufferfish)	ovary: 125 MU·g^−1^	*Bacillus* sp.	+	HPLC, EMI-MS
[16]	*Takifugu obscurus* (pufferfish)	ovary: N/A	*Aeromonas* sp.	1.88 μg·L^−1^ cultured bacteria	ELISA
[21]	*Arothron hispidus* (pufferfish)	1 μg·g^−1^	*Vibrio harveyi*	0.05–1.57 μg·mL^−1^	LC-MS
[15]	*Nassarius semiplicatus* (sea snail)	2 × 10^2^ MU·g^−1^ tissue (3.6 mg in 100 g tissue)	*Vibrio* spp.	11–184 ng·g^−1^	competitive ELISA
			*Marinomonas* spp.	85–98 ng·g^−1^	competitive ELISA
			*Tenacibaculum* spp.	54 ng·g^−1^	competitive ELISA
[8]	*Pseudocaligus fugu* (copepod)	N/A	*Roseobacter* sp.	+	HPLC, GC-MS, LC-MS
[23]	*Chelonodon patoca* (pufferfish)	skin: N/A	*Serratia marcescens*	+	HPLC
[18,19]	*Fugu rubripes* (pufferfish)	ovary: 120 ± 6.2 MU·g^−1^	*Bacillus* spp.	0.1–1.6 MU·g^−1^ cells	mouse bioassay
			*Nocardiopsis dassonvillei*	0.5 MU·g^−1^ cells	mouse bioassay
			*Actinomycete* spp.	0.1–1.6 MU·g^−1^ cells	mouse bioassay
[17]	*Takifugu alboplumbeus* (pufferfish)	intestines: 24.9 ± 24.2 MU·g^−1^ [24]	*Vibrio* spp.	78.3 MU in 500 mL broth (4 × 10^7^ cells)	mouse bioassay
	*Takifugu niphobles* (pufferfish)	ovary—100–1000 MU·g^−1^ [24]	*Microbacterium arabinogalactanolyticum*	105.3 MU in 500 mL broth (4 × 10^7^ cells)	mouse bioassay
[25]	Seven species of nemertean worms	N/A	*Vibrio* spp.	+	HPLC
[13]	*Fugu vermicularis radialis* (pufferfish)	70 ± 8 MU·g^−1^	*Vibrio* spp.	+	HPLC
[26]	*Meoma ventricosa* (sea urchin)	N/A	*Pseudoalteromonas* spp.	+	immunoassay
[11]	*Niotha clathrata* (marine gastropod)	2–50 MU·g^−1^	*Vibrio* spp.	+	HPLC
			*Pseudomonas* spp.	+	HPLC
			*Aeromonas* spp.	+	HPLC
			*Plesiomonas* spp.	+	HPLC
[27]	Freshwater sediment	+HPLC, GC-MS	*Micrococcus* spp.	+	HPLC
			*Bacillus* spp.	+	HPLC
			*Caulobacter* spp.	+	HPLC
			*Flavobacterium* spp.	+	HPLC
[28]	Marine sediment	+HPLC, GC-MS	*Streptomyces* spp.	+	HPLC
[29]	Deep sea sediment	25–90 ng TTX equivalents g^−1^ of mud [30]	*Vibrio* spp.	+	HPLC
			*Bacillus* spp.	+	HPLC
			*Acinetobacter* spp.	+	HPLC
			*Alteromonas* spp.	+	HPLC
			*Aeromonas* spp.	+	HPLC
			*Micrococcus* spp.	+	HPLC
[31]	Four species of Chaetognaths (arrowworms)	320 pg individual^−1^ [32]	*Vibrio* spp.	280–790 pg·μL^−1^ culture medium	cell culture bioassay
[12]	*Hapalochlaena maculosa* (blue-ringed octopus)	140–174 MU idividual^−1^	*Vibrio* spp.	+	HPLC, GC-MS
			*Pseudomonas* spp.	3 MU, +	mouse bioassay, HPLC, GC-MS
			*Bacillus* spp.	5 MU, +	mouse bioassay, HPLC, GC-MS
			*Alteromonas* spp.	+	HPLC, GC-MS
[33]	*Takifugu niphobles* (pufferfish)	intestine 3890 MU·g^−1^	*Shewanella putrefaciens*	15 MU in 250 mL culture broth, +	mouse bioassay, HPLC, GC-MS
[34]	*Fugu vermicularis vermicularis* (pufferfish)	178 MU·g^−1^	*Vibrio* spp.	3 MU, +, +	mouse bioassay, HPLC, GC-MS
[35]	*Astropecten polyacanthus* (comb seastar)	32 MU·g^−1^	*Vibrio* spp.	+	HPLC, GC-MS
[36]	*Fugu poecilonotus* (pufferfish)	N/A	*Pseudomonas* spp.	+	HPLC, GC-MS
[34]	*Atergatis floridus* (reef crab)	+ TLC, eletrophoresis	*Vibrio* spp.	+	HPLC, GC-MS
[7]	*Jania spp.* (red alga)	N/A	*Pseudomonas* spp.	+	HPLC, GC-MS

* MU: Mouse Units; HPLC: high-performance liquid chromatography; GC-MS: gas chromatography-mass spectrometry; TLC: thin layer chromatography; EMI-MS: Electrospray ionization-mass spectrometry; ELISA: enzyme-linked immunosorbent assay; LC-MS: liquid chromatography-mass spectrometry; ** “+”: Denotes positive detection but no quantitative information given.

Research on terrestrial TTX-containing organisms has found limited evidence to support exogenous sources of TTX and endogenous production is commonly postulated. Lehman *et al.* [37] were unable to PCR amplify 16S ribosomal RNA (rRNA) genes using bacterial specific primers from toxic tissues of the rough skinned newt (*Taricha granulosa*), including the liver, gonads, and skin. Positive amplification was obtained from intestines; however, TTX concentrations in these tissues were consistently low. Additionally, when *T. granulosa* were induced via electrical stimulus to excrete TTX through their skin, TTX concentrations were found to regenerate after nine months in captivity, despite being maintained on a TTX-free diet [38]. Collectively these studies indicate that symbiotic bacteria are unlikely to be the source of TTX in this species.

In 2009, populations of the opisthobranch *Pleurobranchaea maculata* (grey side-gilled sea slug; Family: Pleurobranchidae) from Auckland (New Zealand) were found to contain significant concentrations of TTX [39]. Located in shallow sub-tidal areas they are known to be opportunistic scavengers with diets including algae, mussels and anemone [40]. Recent studies have revealed distinct spatial patterns in TTX concentrations among populations with specimens from the South Island containing no detectable TTX [41]. It has also been suggested that the high concentrations of TTX measured in adults during the egg laying season (June–August) and in eggs and early larval stages, indicates that *P. maculata* utilize TTX for protection and to increase survival rates of their progeny [41]. In 2013, high concentrations of TTX were detected in a Platyhelminthes *Stylochoplana* species from Pilot Bay (Tauranga, New Zealand), a site where toxic *P. maculata* occur [42]. Similar seasonal trends were shown in the *Stylochoplana* sp. population and preliminary studies on TTX in egg masses suggest that the toxin could also play a protective role in this species. Salvitti *et al.* [42] used molecular techniques to probe the foregut contents of *P. maculata* and demonstrated that they consume *Stylochoplana* sp. However, based on the concentrations of TTX in *Stylochoplana* sp. and *P. maculata*, and probable growth and consumption rates it is unlikely that they are their only supply of TTX. The co-occurrence of these species may indicate that they are both sourcing TTX from the same dietary source. A microbial origin (either dietary or endosymbiotic) of TTX (or a precursor molecule) is highly likely, given that extensive environmental surveys of hundreds of organisms at sites with dense populations of highly toxic *P. maculata* only detected trace (<0.1 mg·kg^−1^) quantities of TTX in a few organisms [43].

Chau *et al.* [44] recently isolated a limited number (16 isolates, 9 strains) of bacteria from adult *P. maculata* and found no evidence of TTX production. Multiple researchers have suggested that microbial organisms may produce a precursor molecule which is then converted to TTX through a yet-to be identified biochemical pathway within the host organisms [25,45]. This could explain why TTX was not detected in *P. maculata* isolates previously and/or why only low concentrations have been shown to be produced by other bacteria. McNabb *et al.* [46] recently developed an LC-MS method to detect the carbon backbone of TTX. This method detects TTX precursor or degradation products that form the C9 base (2-amino-6-(hydroxymethyl)quinazolin-8-ol) of TTX under the reaction conditions described. The method will not detect all potential molecules related to TTX and will exclude some newly discovered analogues [47]. However as the C9 base reaction is the basis of HPLC detection this method will at least detect anything previously assigned to TTX by HPLC. This is the first study to utilize this method to screen bacterial isolates for the C9 base of TTX.

The aim of this study was to utilize standard microbiological methods, similar to those used in previous studies, to attempt to isolate TTX-producing bacteria from *P. maculata* and *Stylochoplana* sp. [16,25,48]. In 2013, three *P. maculata* and three *Stylochoplana* sp. from Pilot Bay, New Zealand were collected and aseptically dissected. Over 100 bacterial strains were isolated on three different media types under aerobic conditions. To determine the diversity of the strains a region of the 16S rRNA gene was PCR amplified and the products were analyzed by restriction digest analysis. Representatives of each unique banding pattern were sequenced, grown in batch culture and analyzed for the C9 base using LC-MS. Researchers have suggested that the significantly lower concentrations of TTX produced by isolates *in vitro* may be due to the lack of an “inducer” provided by either the host organism or associated bacterial community [5,49]. To explore the possibility that TTX is produced by a consortium of bacteria or influenced by host tissues, a series of broth experiments were also undertaken. These involved inoculating marine broth with subsamples of organs/tissue from *P. maculata* and *Stylochoplana* sp. and tracking TTX concentrations over a series of days.

## 2. Results and Discussion

### 2.1. Bacterial Isolation and Toxin Analysis

All of the tissue samples from individual *P. maculata* and *Stylochoplana* sp. tested positive for TTX via LC-MS (Table 2). Tetrodotoxin concentrations in *P. maculata* specimens from Matakana Island were low when compared to those reported in populations from near-by Pilot Bay (ave. 90 mg·kg^−1^; [41,42]). The individuals were collected in May, before known peaks in TTX occur (June–August), which may partially explain their unusually low TTX concentrations [41]. Additionally, it is possible that the individuals used in this study had not consumed any *Stylochoplana* sp., a suggested dietary source of TTX for this species [42]. The concentrations detected still indicate that they may have accessed (or harbored) an alternative and possibly microbial source of TTX. Thus, it was deemed reasonable to continue isolating bacteria from these individuals. In contrast, TTX concentrations of *Stylochoplana* sp. (ave. 174 mg·kg^−1^) were consistently in the range of previously sampled specimens (ave. 380 ± 210 mg·kg^−1^; [42]).

**Figure 1:**
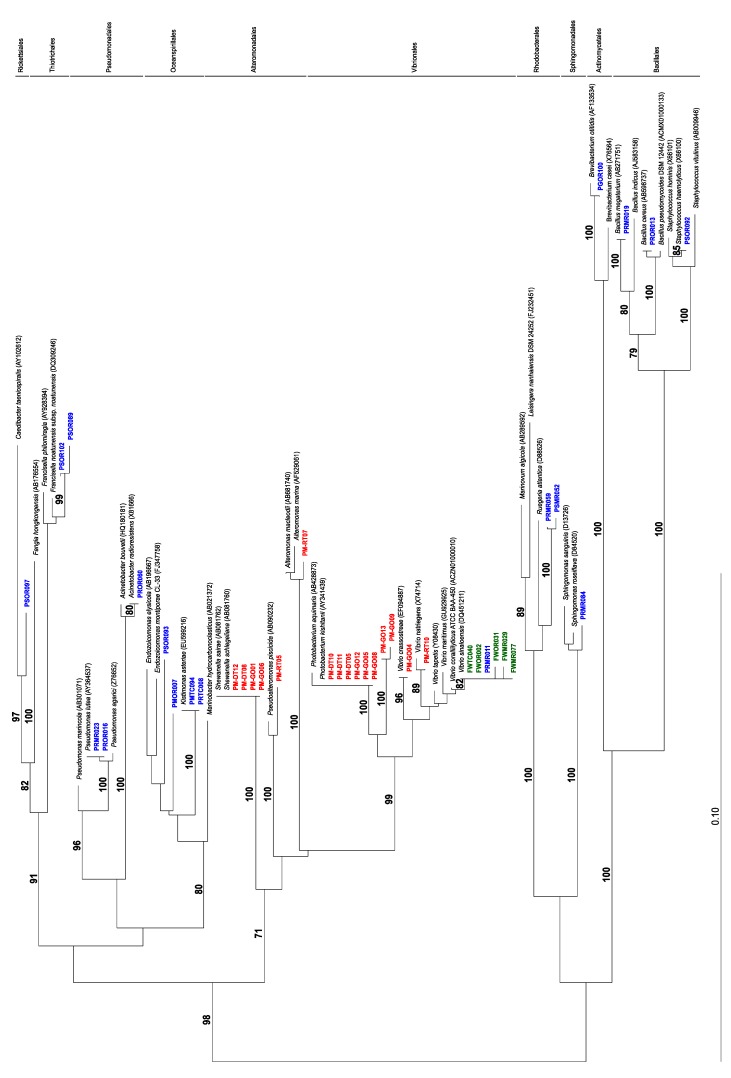
Neighbor-joining phylogenetic tree of 16S rRNA gene sequences of isolates from this study and related bacteria. Isolates from different organisms are color coded as follows: green = *Stylochoplana* sp., blue = *Pleurobranchaea maculata* (this study), red = *P. maculata* [44]. (Bootstrap values < 70 are omitted).

A total of 102 bacterial strains were isolated from the 5 samples (*P. maculata*-63; *Stylochoplana* sp.-39) and their diversity was assessed by restriction fragment length polymorphism analysis (RFLP) of a region of the 16S rRNA gene (Table 2). This analysis identified 28 unique strains or operation taxonomic units (OTUs). Sequencing of the 16S rRNA gene from a representative isolate of each OTU yielded 16 unique strains from *P. maculata* tissues and one from *Stylochoplana* sp. tissues. Phylogenetic analyses revealed that the *P. maculata* 16S rRNA gene sequences grouped into eight distinct clades representing the orders: *Pseudomonadales*, *Actinomycetales*, *Oceanospirillales*, *Thiotrichales*, *Rhodobacterales*, *Sphingomonadales*, *Bacillales*, and *Vibrionales*, whereas the *Stylochoplana* sp. sequences grouped into one clade representing *Vibrionales* (Figure 1). One *P. maculata* isolate PRMR011, grouped phylogenetically with the isolates from *Stylochoplana* sp. Chau *et al*. [44] sequenced the 16S rRNA gene from ten different bacterial strains isolated from the tissues of adult *P. maculata* collected from Narrow Neck Beach (Auckland, New Zealand). Their sequences grouped into two different clades representing the orders *Alteromonadales* and *Vibrionales* (shown in red—Figure 1). In this study, strains representing an additional seven clades have been isolated. These differences could possibly be due to the individuals having been collected from different geographic locations, or having consumed a different dietary source prior to sampling, and further sampling and bacterial isolation efforts from multiple populations are required to establish the degree of variability in microbial consortiums among sites.

**Table 2:** Concentrations (mg·kg^−1^) of tetrodotoxin (TTX) and number of bacterial strains isolated from pooled (*n* = 3) tissues samples of *Pleurobranchaea maculata* and *Stylochoplana* sp. collected 7 May 2013 from Matakana Island (M.I.) and Pilot Bay (P.B), New Zealand, and TTX concentrations in pooled samples of *P. maculata* tissue used for broth experiments collected from Illiomama Rock (I.R) (Auckland), New Zealand collected 28 September 2011.

Sample	Location	TTX (mg·kg^−1^)	Bacterial Strains
*P. maculata*; digestive	M.I.	2	16
*P. maculata*; gonad	M.I.	5	3
*P. maculata*; mantle	M.I.	7	21
*P. maculata*;“rest”	M.I.	8	23
*Stylochoplana* sp.	M.I., P.B.	174	39
*P. maculata*; digestive	I.R.	771	-
*P. maculata*; gonad	I.R.	136	-
*P. maculata*; mantle	I.R.	97	-

Although many bacteria isolated grew from the *Stylochoplana* sp. inoculum, the molecular analysis showed that the diversity was very low (only one strain). This may indicate that this bacterial species (*Vibro* sp.) is very abundant, or alternatively that other bacterial strains could not grow on the media used in this study, possibly due to an antimicrobial interaction. Pryosequencing could be used to help elucidate the total bacterial diversity within this organism. The *Vibro* sp. strain isolated from *Stylochoplana* sp. was also detected in *P. maculata*, and although no TTX was identified, strains found in both species may be good candidates for further investigation.

To date, eleven studies have isolated a TTX-producing *Vibrio* sp. [11,12,13,15,17,25,29,31,34,35,50] making this the most common genera to be associated with TTX production. Other common groups associated with TTX production include *Bacillus* spp. [12,18,19,22,27,29,51], *Pseudomonas* spp. [7,11,12,36], *Aeromonas* spp. [11,16,29], and *Alteromonas* spp. [12,29]. Although representatives of three of these genera were isolated in this study, phylogenetic comparisons are challenging as only four previous studies that have attempted to isolate TTX-producing bacteria have undertaken any molecular analysis and submitted these data to public databases [15,26,44,51].

To date, the majority of studies describing TTX-producing bacteria have only provided positive detection of toxin from isolated strains, without measuring quantitative concentrations of TTX produced (Table 1). Of those that have provided quantitative concentrations, strains have been shown to produce very low TTX concentrations compared to host organisms. For example, Wu *et al.* [18,19] isolated TTX-producing bacterial strains from the tissues of pufferfish *Fugu rubripes* including the ovaries (120 MU·g^−1^), liver (78.5 MU·g^−1^) and intestines (36.2 MU·g^−1^). In contrast, the toxicity concentrations in bacterial isolates were only 0.1–1.6 MU·g^−1^ of cells. Researchers have suggested that the relatively low concentrations of TTX-producing bacterium are due to the altered conditions when grown *in vitro* or, alternatively, that strains are providing hosts with TTX precursors. Thus, in this study we tested bacterial strains for the C9 base of TTX using the methods described in McNabb *et al.* [46] as it may detect TTX precursors or degradation products. It also has the additional benefit of greater sensitivity with the limit of detection. *ca.* 0.1 mg·kg^−1^ compared to the standard TTX LC-MS-based method used by our research group of. *ca.* 0.5 mg·kg^−1^ [39]. Despite the additional benefits and sensitivity of this method no C9 base was detected in any samples (data not shown).

### 2.2. Bacterial Community Analysis

**Figure 2:**
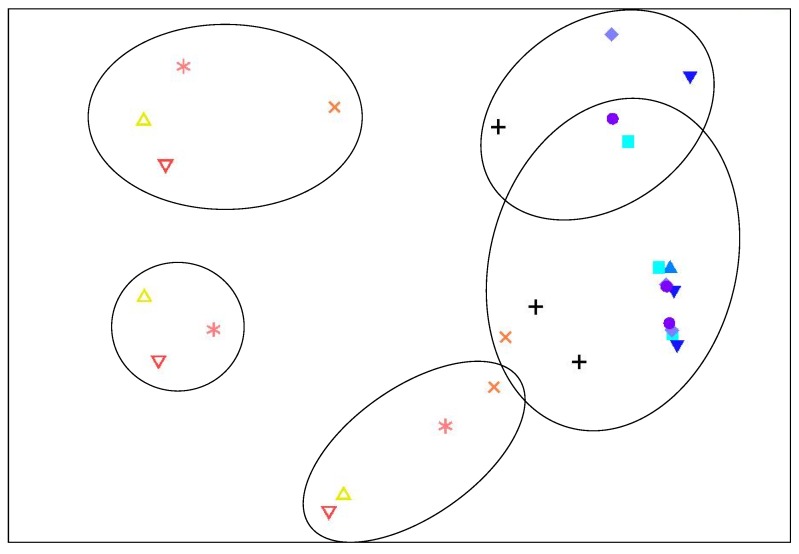
Two-dimensional non-metric multidimensional scaling ordination based on Bray-Curtis similarities of Automated Ribosomal Intergenic Spacer Analysis (ARISA) fingerprints of bacterial communities at different time points in broth experiments (stress = 0.1). 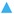 0 days, 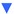 day 1, 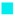 day 2, 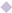 day 3, 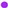 day 4, + day 6, 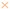 day 8, 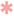 day 10, 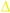 day 12, 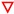 day 14. Points enclosed by solid line cluster at 40% similarity.

The *P. maculata* used to initiate the broth experiments all contained TTX although the concentrations varied considerably (Table 2). One limitation of this experiment is that specimens were maintained in aquarium for up to five days prior to dissection and inoculation of the broths. It is possible that TTX-producing bacteria may have expired or been expelled during this period. However, given the considerable concentration of TTX in the individuals (Table 1), we suggest that any TTX-producers would have been present in high concentration, and therefore it is unlikely that there would be none remaining. These samples were pooled by tissues type prior to initiation of the broth experiments. Marine broth samples from day 0 had trace levels of TTX (data not shown). No TTX was detected in the day 3, 6, and 10 samples. The multidimensional scaling (MDS) analysis of the bacterial communities as determined using ARISA showed a 40% similarity between those samples taken in the first four days indicating limited change in the community structure and abundance of each strain among those time points (Figure 2). By day 4 the broths all contained dense bacterial assemblages (as determined by the cloudy nature of the broth), thus, if a TTX- producing bacterium were present in the intimal inoculum it should have had sufficient time to produce toxins before possibly being outcompeted by other bacteria within the community.

Using standard microbiological methods, and very similar media and conditions to studies describing the successful isolation of many TTX producing bacteria no TTX-producing isolates were identified from either *Stylochoplana* sp. or *P. maculata* in this study. By using a new method that detects the C9 base of TTX, we had anticipated that the possibility of detecting precursor or degradation molecules would be increased, however, none were detected. The biosynthetic pathway of TTX is unknown, thus it is possible that not all precursors would be detected via this method. Another possibility is that an “unculturable” bacterium might produce TTX. Many studies have now shown that less than 1% of bacteria within a particular community are culturable [52]. However, based on previously published research (Table 1), many of the TTX-producing strains are genera which can be easily cultured. Strains isolated in this study fall into four of these genera, yet no C9 base of TTX was detected in any of the isolates, or microbial communities, suggesting that further efforts to isolate TTX-producing bacterium might not be warranted. Among this literature extensive culturing efforts are not reported (*i.e*., generally less than 50 strains are isolated) to identify a TTX-producer and there are few [to our knowledge one; 44] that report unsuccessful attempts to isolate TTX-producing strains.

## 3. Experimental Section

### 3.1. Bacterial Strain Isolation

#### 3.1.1. Collection and Strain Isolation

Collections took place (7 May 2013) from two sites in Tauranga Harbor, New Zealand. Three *P. maculata* and two *Stylochoplana* sp. were collected by divers from Matakana Island (37°38'38" S, 176°8'55" E) and an additional *Stylochoplana* sp. specimen from Pilot Bay (37°63'5'' S, 176°17'6'' E). Specimens were transported to the laboratory in insulated containers and placed in aerated aquaria overnight before being rinsed with deionized water. *Pleurobranchaea maculata* were aseptically dissected and separated in to four tissue types; gonad, digestive organs, mantle and remaining tissues (“rest”). Each of the four tissue types from the three individuals were combined and homogenized to give four samples. The three *Stylochoplana* sp. specimens were combined and homogenized into one sample. Subsamples from each were frozen (−20 °C) for later toxin analysis. Aliquots of the five combined samples were diluted 1:10 (*w/v*) in marine broth (Difco), manually homogenized using a glass pestle, and centrifuged (1000× *g*, 1 min). The supernatant was then diluted (100, 1000, and 10,000 fold) and 50 μL aliquots were used to inoculate three types of agar which have previously been used to isolate TTX-producing bacteria; marine agar 2216 (Difco), Thiosulfate Citrate Bile Salts Sucrose TCBS agar (Difco), and Ocean Research Institute (ORI) agar [10,53]. Agar plates were placed in an incubator (*ca.* 20 °C) and grown for up to nine days. Multiple representatives of individual colonies that differed in morphology were selected. To ensure that each culture was comprised of a single strain, each was streaked again onto marine agar 2216 (Difco) and grown for 2–3 days at 27 °C. Single colonies were collected, grown overnight (27 °C) in marine broth 2216 (Difco), and stored frozen (−20 °C) after being split into two tubes with the following treatments; (1) preserved with 15% sterile glycerol and stored at −80 °C for later culturing, and (2) centrifuged (10,000× *g*, 10 min) with the supernatant removed for later DNA extraction.

#### 3.1.2. Molecular and Phylogenetic Analysis of Bacterial Strains

DNA was extracted from bacterial pellets using a prepGEM^®^ DNA Bacterial Extraction Kit (Zygem, Hamilton, New Zealand) according to the manufactures instructions. The PCR of bacterial 16S rRNA genes was performed using the primers 27F (5'-AGAGTTTGATCMTGGCTCAG-3') and 1518R (5'-AAGGAGGTGATCCANCCRCA-3'). Reactions were carried out in 25 µL volumes with the reaction mixture containing; 2.5 µL of 10×PCR buffer (Invitrogen, Waltham, MA, USA), 1.3 mM MgCl_2_ (Invitrogen, USA), 0.2 mM (each) de-oxynucleoside triphosphate (Bioline, Taunton, MA, USA), 0.02 mg·mL^−1^ bovine serum albumin (BSA, Sigma, St. Louis, MO, USA), 0.25 μM of each primer (IDT, San Diego, CA, USA), 0.04 U of Platinum *Taq* DNA polymerase (Invitrogen, USA), and 20–30 ng of template DNA. The reaction mixture was held at 94 °C for 2 min followed by 30 cycles of 94 °C for 20 s, 57 °C for 20 s, 72 °C for 1 min, with a final extension of 72 °C for 7 min. The resulting PCR products were screened by restriction fragment length polymorphism (RFLP) patterns generated using the restriction endonuclease HaeIII (as per the manufactures instructions) and based on their banding patterns grouped into operational taxonomic units (OTUs). One representative of each OTU was sequenced using the BigDye Terminator v3.1 Cycle Sequencing Kit (Applied Biosystems, Waltham, MA, USA) on a ABI3100 (Applied Biosystems, Waltham, MA, USA) using the 27F primer. Sequences obtained in this study were deposited in the NCBI GenBank database under accession numbers KJ995704 to KJ995726. Phylogenetic analysis of isolates was conducted by aligning their sequences, and those from Rocky *et al.* [44], to closely matching sequences from the Greengenes [54] database of bacterial 16S sequences using ARB [55]. Aligned sequences were 399 bp in length and all gaps and ambiguities were excluded from the alignment to ensure reliability. Phylogenetic inferences were made using the PHYLIP package [56]. Pairwise evolutionary distances were computed from percent similarities by the correction of Jukes and Cantor [57] and the phylogenetic tree was constructed by the Neighbor-joining method [58]. The support for each node was determined by assembling a consensus tree of 1000 bootstrap replicates.

#### 3.1.3. Bacterial Culturing

One representative of each unique bacterial strain (as identified using RFLP) was retrieved from the cryopreserved stocks, inoculated into marine broth (400 mL) and grown at 30 °C with shaking (110 rpm) for 4 days. Cultures were centrifuged (6000× *g*, 20 min) and the supernatant removed and the pellets frozen (−20 °C) for later C9 analysis (Section 3.3).

### 3.2. Bacterial Community Broth Experiments

#### 3.2.1. Collection and Inoculation

Three *P. maculata* were collected by divers (28 September 2011) from Illiomama Rock (36°48'44"S, 174°52'48"E), Auckland Bay, New Zealand. The specimens were transported to the laboratory in insulated containers and placed in aquaria for five days. *Pleurobranchaea maculata* were aseptically dissected and separated into three tissue types; gonad, digestive organs and mantle. Each of the three tissue types from the three individuals were combined, homogenized (1 min, Heidolph Diax 600 Homogenizer, Heidolph, Germany) and diluted 1:10 (*w/v*) in marine broth (Difco). Samples were then homogenized (1 min, Heidolph Diax 600 Homogeniser) to ensure that bacteria from the tissues were dispersed throughout the media, and then centrifuged (1000× *g*, 1 min) to prevent tissue being inoculated into the broths. Aliquots (200 μL) of each supernatant were added to separate 1-L marine broth (Difco) in Erlenmeyer flasks. Three control flasks were used. A second contained marine broth spiked with TTX (Tocris Bioscience, Cat. No: 1078) at a final concentration of 6.9 μg·mL^−1^ (to ensure that there was no TTX degradation over the experimental period). This concentration was chosen as it could easily be detected allowing changes in TTX to be monitored throughout the experiment. A final control consisting of marine broth, mantle tissue supernatant and sodium azide (0.02% final volume *w/v*) was used to ensure that any increase in TTX was not due to TTX-unbinding from tissue, or a similar scenario causing an increase in toxins. Flasks were placed in a thermally controlled shaker (120 rpm) set approximately at 25 °C. Sub-samples for TTX analysis (30 mL) and DNA extraction (1 mL) were collected on day 0, 1, 2, 3, 4, 6, 8, 10, 12, and 14. These were centrifuged (3000× *g*, 10 min) and the supernatant removed before the remaining pellets were stored frozen (−20 °C) for later TTX and molecular analysis.

#### 3.2.2. Molecular Analysis

DNA was extracted from the broth experiment pellets using hexadecyl trimethyl-ammonium bromide (commonly known as the CTAB method) as described in Barrett *et al*. [59]. Automated rRNA intergenic spacer analysis (ARISA) is a PCR-based method that exploits the length heterogeneity of the intergenic spacer region (ITS) between the 16S and 23S ribosomal genes. Total community DNA is amplified with a fluorescently labeled forward oligonucleotide, allowing the electrophoretic step to be performed with an automated system in which a laser detects the fluorescent PCR fragments, providing a “finger-print” of the bacterial community in each sample. In this study, ARISA was used to track shifts in bacterial community structure throughout the broth experiments, with the aim of determining if these were associated with changes in TTX concentrations. Polymerase chain reactions for ARISA were preformed using the reaction mixture described above and the bacterial primers ITSF and ITSReub from Cardinale *et al.* [60]. Reactions were run on an DNAEngine^®^ Peltier thermal cycler (Biorad, Hercules, CA, USA) with the following cycling parameters: 94 °C for 2 min, 30 cycles of 94 °C for 45 sec, 55 °C for 60 sec, 72 °C for 2 min, and a final extension of 72 °C for 7 min. PCR products were visualized on 1% agarose gel and then diluted 20 fold using Milli-Q water. Intergenic spacer fragments were run on an ABI 3130 xI sequencer (PE Applied Biosystems, Foster City, CA, USA) employing the GeneScan mode at 15 kV for a run time of 45 min according to the manufacturer’s manual. The internal GS1200LIZ Zy Standard (0.25 µL; PE Applied Biosystems) was added to each sample to determine the size of fluorescently labelled fragments during analysis. PeakScanner™ software v1.0 (PE Applied Biosystems) and an in-house pipeline modified from Abdo *et al.* [61] written using Python 2.7.1 (Python Software Foundation) and R [62] were used to process ARISA profiles. Electropherogram analysis included all peaks that made up 0.1% of the entire signal, were between 100 and 1200 base pairs, and were over 30 relative fluorescence units. Peaks were binned to the nearest 1 base pair. ARISA fluorescence intensities data were log transformed and analyzed with the PRIMER 6 software package (PRIMER-E, Ltd., Plymouth, UK) using nonmetric multidimensional scaling (MDS) based on Bray-Curtis similarities conducted with 100 random restarts. Results and agglomerative hierarchical clustering of similarities, executed using the CLUSTER function, were plotted onto two-dimensional plots.

### 3.3. Tetrodotoxin and C9 Analysis

Tissue samples from *P. maculata*, *Stylochoplana* sp., and pellets from broth aliquots from day 0, 3, 6, 8, and 10 were extracted using a slightly modified method from McNabb *et al*. [39]. Milli-Q water containing 0.1% acetic acid was added on a 1:10 *w/v* basis to sub-samples of tissue or cell pellet and homogenized (for tissue; Heidolph Diax 600 Homogeniser; Heidolph, Germany) or sonicated (for cell pellet; Misonix XL2020, Misonix Inc., Farmingdale, NY, USA). Samples were centrifuged (3000× *g*, 10 min) and an aliquot of the supernatant (1 mL) transferred into 9 mL of 100% methanol containing 0.1% acetic acid and placed at −20 °C for at least 1 h. After freezing, samples were centrifuged (3000× *g*, 10 min) and diluted 1:4 with 100% methanol containing 0.1% acetic acid. Samples were analyzed for TTX using LC-MS as described in McNabb *et al.* [39].

Frozen bacterial isolate pellets for testing for the C9 base were extracted using methods from McNabb *et al.* [46]. Briefly, Milli-Q water with 0.1% acetic acid was added to. *ca.* 1.0 g pellet on a 1:10 *w/v* basis. Samples were then homogenized using a sonicator (Heidolph Diax 600 Homogeniser; Heidolph, Germany) set at level 4 for 30 s and centrifuged (3000× *g*, 5 min). Supernatant was transferred to a new tube and sodium hydroxide was added to a final concentration of 1 M. Tubes were then placed in a boiling water bath (45 min), cooled, and neutralized with concentrated acetic acid to pH 4–6. Samples were purified and concentrated using an SPE cartridge (Phenomonex StrataX; 60 mg 3 mL^−1^). These were conditioned with methanol (MeOH, 100%; 3 mL) followed by 50 mM ammonium acetate (3 mL). After samples were loaded, the filter was washed with 50 mM ammonium acetate (3 mL), followed by 5% MeOH in 50 mM ammonium acetate (3 mL). Samples were eluted (3 mL) using of 30% MeOH containing 1% formic acid, and tested for the C9 base via LC-MS as described in McNabb *et al.* [46].

## 4. Conclusions

This study used a recently developed highly sensitive LC-MS based method to attempt to identify the C9 base or TTX precursor/degradation products in bacterial strains isolated from toxic *P. maculata* and *Stylochoplana* sp. A total of 102 strains were isolated and sequencing of the 16S rRNA gene from a representative isolate of each OTU yielded 16 unique strains from *P. maculata* tissues and one from *Stylochoplana* sp. tissues. Despite these intensive culturing efforts, newly developed extremely sensitive TTX detection capabilities, and an experiment where we investigated whether a consortium of bacteria from *P. maculata* could produce TTX, no evidence was found to support a bacterial origin of TTX in *P. maculata* or *Stylochoplana* sp.
